# The So-Called Lunacy "Scandals"

**Published:** 1900-07-21

**Authors:** 


The Hospital

A Journal of

The Medical Sciences and Hospital Administration.

Vol. XXVIII.—No. 721.] Edited by Sir Henry Burdett, K.C.B., and Solomon C. Smith, M.D., M.R.C.P. [July 21, 1900.

### THE SO-CALLED LUNACY "SCANDALS."

Almost coincidently with our recent observations upon the controversy with regard to the treatment of insanity, which is in progress in the State of New York between asylum superintendents and scientific pathologists, it was announced in the London papers that a certain number of the members of the London County Council proposed to visit the United States at the expense of the ratepayers, and with the declared object of contrasting the English and the American methods of dealing with the subject. It is worthy of remembrance that one of the most important improvements in the treatment of the insane which have been made in this country during the present century was mainly due to the almost accidental appointment, as Medical Superintendent of Hanwell, of a physician, the late Dr. Conolly, who was destitute of the particular qualification upon which visiting justices and others have been wont chiefly to lay stress, the qualification of "asylum experience." In other words, his mind had not been case-hardened by routine; and he was shocked by practices which routine declared to be necessary, and which he at once showed, by abandoning them, to be not only unnecessary, but in a high degree injurious. His appointment was but a transient gleam of sunshine upon one of the darkest of human prospects, for, on his retirement, the demand for "asylum experience" again became predominant, with the natural result that everyone engaged in the administration of the "system" was forced into its grooves at a comparatively early period of life, having done no work as a physician except under its paralysing influence. The result has been to develop a class of acute administrators, capable disciplinarians, and shrewd managers, whose attention has been systematically diverted from the salient fact of the case, the fact that insanity is as much a bodily disease as phthisis or pneumonia, a disease depending upon changes in the nervous system which, in all probability, might be detected in their incipient stages by a trained pathologist, and which might perhaps be arrested, and even made to issue in complete recovery, by a trained clinical physician.

In the meanwhile, and by another curious coincidence, we have the investigation by the Local Government Board of the recent disclosures in the parish of St. Pancras—disclosures which are described as "scandals" by some of our non-medical contemporaries, to whom every undetected pickpocket is a "mystery," and every overturned wheelbarrow an "appalling phenomenon." The "scandals" seem to amount to this, that it has been left to the relieving officers to select the medical man by whom a pauper supposed to be a lunatic should be certified, and to select also the asylum to which he or she should be sent at the cost of the parish. The certifying doctor is, of course, paid for his work; and the proprietors of the asylum are paid a weekly sum, on which they presumably make a profit, for the maintenance of the insane person. The pay and the profits have been shared, by some doctors and by some asylum keepers, with the relieving officers to whom they were primarily indebted for them; with the result, apparently, that certain parish doctors have been deprived of their fair share of certifying fees, and certain asylums of their fair share of inmates, with the addition that, in some cases, the asylums so deprived were less expensive to the ratepayers than those to which the unfortunates concerned were actually consigned. It is obvious that all this is very rotten, and very bad, that the discretion vested in relieving officers has been one which they were not fit to bear, that no such "patronage" should be left in their hands, and that every parish doctor, as a matter of right, should give all the certificates which are required within his district. But as to the choice of an asylum, we see no reason to believe either that the Board of Guardians would be any better qualified than the relieving officer, or that they would be influenced by any other consideration than the amount of the weekly payment. They would, of course, have no temptations as regards their own pockets, but would think first, and probably only, of the greater or less cost to the ratepayers, and the destination of the lunatic, however honestly decided upon, would turn upon the question of money and of cost rather than upon that of the treatment he requires. It is manifest that, under a scientific system, or even under a rough division of cases into the curable and the incurable, a great room for salutary choice might exist; but the power of exercising it in a proper manner could not safely be committed to ignorance, whether flourishing in the relieving office, where it might be tempered by love of pocket, or in the board-room, where it might be guided by care for rates. It should be a primarily medical function; and it will become so as soon as the essentially morbid character of insanity is recognised, and the natural results follow from the recognition. As long as people talk about "*diseases* of the *mind*," so long, presumably, "porochial" authorities will retain control over the sufferers, and affairs will continue to drift in the present unsatisfactory and well-nigh hopeless fashion.

